# CENP-A binding domains and recombination patterns in horse spermatocytes

**DOI:** 10.1038/s41598-019-52153-1

**Published:** 2019-11-01

**Authors:** Eleonora Cappelletti, Francesca M. Piras, Claudia Badiale, Marina Bambi, Marco Santagostino, Covadonga Vara, Teri A. Masterson, Kevin F. Sullivan, Solomon G. Nergadze, Aurora Ruiz-Herrera, Elena Giulotto

**Affiliations:** 1 Department of Biology and Biotechnology “Lazzaro Spallanzani”, University of Pavia, 27100 Pavia, Italy; 2 Departament de Biologia Cellular, Fisiologia i Immunologia, Universitat Autònoma de Barcelona, Cerdanyola del Vallès, 08193 Spain; 3 Genome Integrity and Instability Group, Institut de Biotecnologia i Biomedicina, Universitat Autònoma de Barcelona, Cerdanyola del Vallès, 08193 Spain; 4 Centre for Chromosome Biology, School of Natural Sciences, National University of Ireland, Galway, Ireland

**Keywords:** Centromeres, DNA recombination, Meiosis

## Abstract

Centromeres exert an inhibitory effect on meiotic recombination, but the possible contribution of satellite DNA to this “centromere effect” is under debate. In the horse, satellite DNA is present at all centromeres with the exception of the one from chromosome 11. This organization of centromeres allowed us to investigate the role of satellite DNA on recombination suppression in horse spermatocytes at the stage of pachytene. To this aim we analysed the distribution of the MLH1 protein, marker of recombination foci, relative to CENP-A, marker of centromeric function. We demonstrated that the satellite-less centromere of chromosome 11 causes crossover suppression, similarly to satellite-based centromeres. These results suggest that the centromere effect does not depend on satellite DNA. During this analysis, we observed a peculiar phenomenon: while, as expected, the centromere of the majority of meiotic bivalent chromosomes was labelled with a single immunofluorescence centromeric signal, double-spotted or extended signals were also detected. Their number varied from 0 to 7 in different cells. This observation can be explained by positional variation of the centromeric domain on the two homologs and/or misalignment of pericentromeric satellite DNA arrays during homolog pairing confirming the great plasticity of equine centromeres.

## Introduction

A key step of meiosis is the recombination between homologous chromosomes, a process that has the dual role of providing immediate physical connections between homologs and increasing genetic diversity^1^. In the leptotene phase of prophase I, homologous chromosomes begin to pair through the assembly of the synaptonemal complex (SC), an evolutionarily conserved zipper-like protein structure^2,3^. This stage is followed by zygotene, where meiotic recombination is triggered by the formation of SPO11 induced double strand breaks (DSBs)^4^. It is later on, in the pachytene phase, when homolog pairing is completed and DSBs are resolved producing crossing over events (CO). CO homeostasis and distribution along chromosomes are tightly regulated^5–7^. In particular, it is well known that at least one CO per chromosome pair is required and that the presence of a CO influences the positioning of other CO events on the same chromosome. This phenomenon is called crossing over interference^8,9^.

A key element for the regulation of CO positioning is the centromere, the chromosomal nucleoprotein structure that is the site of kinetochore assembly required for proper chromosome segregation during cell division.

Despite the evolutionary conservation of centromeric proteins, centromeric DNA sequences are extremely divergent and neither necessary nor sufficient for determining the centromeric function. This paradox is explained by the fact that the centromeric function is epigenetically specified and CENP-A, the histone H3 centromeric variant, is the key marker^10,11^.

The centromere exerts a direct, negative effect on meiotic recombination, both within itself and on the proximal regions^12–15^. This effect, which is conserved across eukaryotes, is known as the “centromere effect”. It was shown that crossover suppression ranges from 5-fold to >200-fold in different organisms^16–24^. The precise mechanisms responsible for this phenomenon are still controversial. One hypothesis is that selective pressure to reduce crossing over near the centromere would be strong. Indeed, recombination events too close to the centromere may disrupt pericentric sister chromatid cohesion, strongly affecting kinetochore functionality^15,24^. Since centromeres are known to reside in a heterochromatic environment, a contribution of heterochromatin in this crossover suppression has been proposed^15,24^. Mammalian centromeres are embedded in wide heterochromatic domains which are typically associated to satellite DNA, but it is still unclear whether the presence of repetitive DNA contributes to the “centromere effect”^24^.

To answer this question, we took advantage of the *Equus caballus* (domestic horse) model system^25^. In this species, the centromere of chromosome 11 (ECA 11) is devoid of satellite sequences while all the other centromeres are satellite-based^26,27^. This peculiar centromere, similarly to several satellite-less centromeres in other species of the genus *Equus*, arose recently during evolution as a result of repositioning^28–30^ which is the movement of centromeric function along the chromosome without marker order variation^31^. Besides centromere movement, the genomes of equids are characterized by an exceptional plasticity regarding retrotransposition^32^ and insertion of nuclear mitochondrial DNA fragments^33^.

We previously isolated and characterized the major horse centromeric satellite DNA family, 37cen^34,35^. The satellite-less centromere of ECA 11 was the first one to be identified and described at the molecular level^26^. Recent studies showed that the position of ECA 11 centromere is not fixed but, in different individuals, it slides within a 500 kb window giving rise to positional alleles^36,37^. These “epialleles” are inherited as Mendelian traits, but their position can slide in one generation being stable during mitotic propagation of cultured cells^37^. The “centromere sliding” phenomenon thus confirms the epigenetic nature of centromeres and proves that centromeric domains are characterized by positional instability^36^.

In the present work we mapped recombination foci along horse chromosome 11 and found that, although its centromere is devoid of satellite repeats, it exerts an inhibitory effect on recombination. We then discovered that double well separated CENP-A binding domains were present at several pachytene bivalents and proposed a model to explain their formation.

## Results

### Distribution of MLH1 foci in male horse meiosis

The distribution of MLH1 foci, a marker of meiotic crossovers, was investigated at pachytene stage by immunofluorescence. We used an anti-MLH1 antibody to label recombination sites, an anti-SCP3 antibody, for the immunostaining of the synaptonemal complex (SC), and a CREST serum, to label all centromeres. Pachytene cells from three different horses were analysed. In Table 1 the mean number of MLH1 foci per cell and the mean number of foci per bivalent are reported. The horse karyotype is characterized by a diploid number of 31 autosomal bivalents, 13 of which metacentric. The total number of autosomal MLH1 foci per cell ranged from 36 to 54 in horse TE, from 38 to 51 in horse PV and from 42 to 63 in horse MP, with an overall mean frequency of autosomal MLH1 foci of 45.30 ± 4.90 per cell. In the 13 metacentric bivalents we detected an average number of 24.12 ± 3.04 foci per cell, with 1.86 ± 0.23 foci per chromosome, while in the 18 acrocentric chromosomes the mean frequency of MLH1 foci per cell was 21.18 ± 2.78, with an average of 1.18 ± 0.15 foci per bivalent (Table 1). These results are in agreement with the previously described frequency of MLH1 foci in horse male meiosis^38^ highlighting the requirement of an “obligatory CO” per bivalent to ensure chromosomal disjunction. MLH1 foci on the XY body were not taken into account, due to their peculiar organization and meiotic behaviour.

**Table 1:** Distribution of total autosomal MLH1 foci in *Equus caballus*. Mean values are reported with their standard deviations.

Horse	Number of cells	Foci per cell	Foci per bivalent	Metacentrics		Acrocentrics	
				Foci per cell	Foci per bivalent	Foci per cell	Foci per Bivalent
TE	39	44.85 ± 4.54	1.45 ± 0.15	23.95 ± 3.12	1.84 ± 0.24	20.90 ± 2.52	1.16 ± 0.14
PV	10	45.30 ± 4.57	1.46 ± 0.15	23.90 ± 3.00	1.84 ± 0.23	21.40 ± 2.32	1.19 ± 0.13
MP	8	47.50 ± 6.85	1.53 ± 0.22	25.25 ± 2.82	1.94 ± 0.22	22.25 ± 4.30	1.24 ± 0.24
total	57	45.30 ± 4.90	1.46 ± 0.16	24.12 ± 3.04	1.86 ± 0.23	21.18 ± 2.78	1.18 ± 0.15

**Figure 1:**
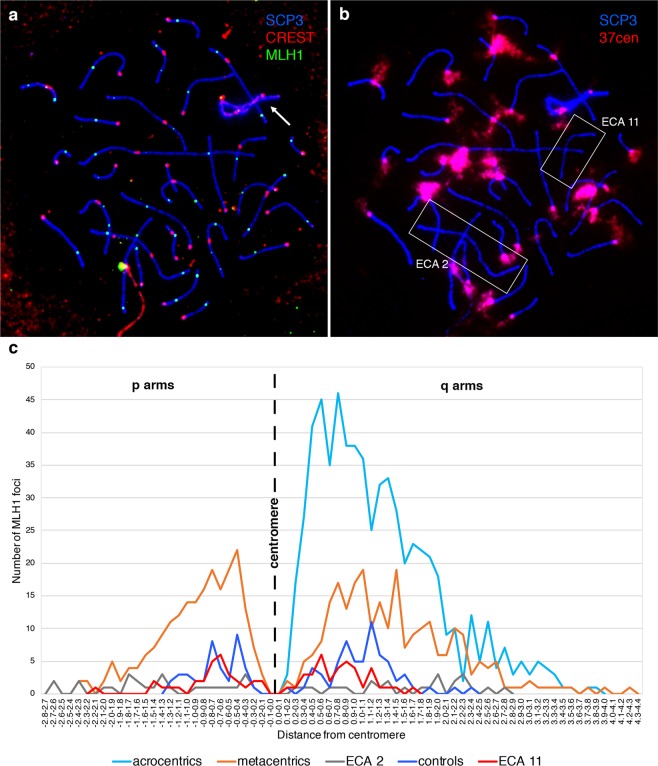
Localization of MLH1 foci. (**a**) Triple immunofluorescence with anti-SCP3 antibody (blue), CREST serum (red) and anti-MLH1 antibody (green) on a horse pachytene spread. The XY body is indicated with an arrow. (**b**) FISH identification of ECA 11 centromere using the 37cen satellite probe (red) on the same pachytene spread. ECA 2 and ECA 11 are the only two chromosomes lacking 37cen signals. ECA 11 can be recognized because it is shorter than ECA 2. (**c**) Distribution on p and q arms of MLH1 foci on ECA 11 (red), control metacentric chromosomes of similar size (blue), ECA 2 (grey), all other metacentrics (orange) and acrocentrics (cyan). The distance from the centromere was measured in centimetres.

To evaluate whether a satellite-less centromere suppresses meiotic recombination at the same level as a satellite-based one, we compared the distance between each MLH1 focus and the centromere on ECA 11 and on all other chromosomes. To this end, we combined immunostaining to localize MLH1 foci and centromeres with FISH using the 37cen satellite DNA as probe. We previously demonstrated that this probe labels all horse centromeres except the ones of ECA 11 and ECA 2^27^. In Fig. 1a, MLH1 foci (green) and centromeres (red) in a pachytene spread are shown and the identification of ECA 11 in the same spread is shown in Fig. 1b. This experimental workflow allowed us to easily identify ECA 11 as the smaller chromosome negative to 37cen hybridization (Fig. 1b). It is important to point out that the centromere of ECA 11 is the only one totally lacking any satellite repeats while, at the centromere of chromosome 2, satellite repeats other that 37cen are present^26,27^. The distance between recombination foci on ECA 11 and the centromere was measured in 42 cells of horse TE, where a total number of 69 MLH1 signals were detected (red line in Fig. 1c). Since the distribution of crossovers is influenced by total chromosomal length^38^, in each one of the 42 spreads, metacentric bivalents with a meiotic length comparable to ECA 11 (length of ECA 11 ± 0.5 cm) were identified as control for a total of 67 synaptonemal complexes and 104 MLH1 foci (blue line in Fig. 1c). This analysis allowed us to compare the distribution of MLH1 foci on ECA 11 and on chromosomes with comparable meiotic length but with satellite-based centromeres. The distance between MLH1 foci and the centromere was also measured on ECA 2, that is the longer chromosome negative to 37cen hybridization (grey line in Fig. 1c). The same measurement was performed on all other metacentrics (orange line in Fig. 1c) and on acrocentric chromosomes (cyan line in Fig. 1c). MLH1 foci on the p and q arms of metacentrics were measured separately.

No MLH1 foci were detected around the centromere region of all bivalents. In particular, the distance between the centromere and MLH1 foci on all metacentrics, including ECA 11, follows a bimodal distribution with a marked depression around the centromere. The distribution of MLH1 foci on the acrocentrics is similar to the one on the q arms of metacentrics with a drop in the frequency of crossovers near the centromere. As expected, the maximum distance covered by the ECA 11 curve (red) and by the curve of the small metacentric chromosomes used as control (blue) is shorter than the one covered by the curves obtained from all other chromosomes. These results show that the centromere effect affects all horse chromosomes independently of the presence of satellite DNA at their centromeres.

### Identification of double and stretched CENP-A signals in horse bivalents

**Figure 2:**
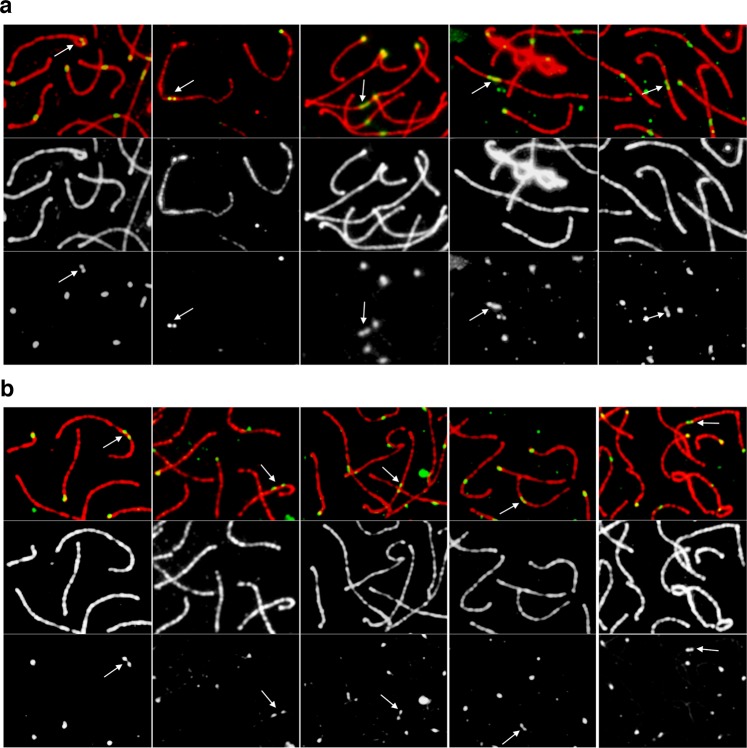
DSS centromeres. (**a**) In the first row, DSS bivalents (white arrows) detected by double immunofluorescence with an anti-SCP3 antibody (red) and a CREST serum (green) are shown. Signals ranged from double-spotted signals to stretched signals (from left to right). In the second and third rows the red and the green channels are shown separately. (**b**) In the first row, DSS bivalents (white arrows) detected by double immunofluorescence with anti-SCP3 antibody (red) and anti-CENP-A serum (green) are shown. Signals ranged from double-spotted signals to stretched signals (from left to right). In the second and third rows the red and the green channels are shown separately.

During the analysis of recombination foci in spermatocytes at the pachytene phase of meiosis we observed a peculiar phenomenon: while, as expected, the centromere of the majority of bivalents was labelled with a single CREST signal, double-spotted centromeres were also detected on several bivalents (Fig. 2a). These signals were never observed on XY bodies.

Besides double signals composed by two well separated dots and canonical single-dotted signals, we could observe “stretched” signals likely deriving from two spots too close to be resolved separately (Fig. 2a). From now on, double and stretched signals will be called DSS (Double and Stretched Signals).

**Figure 3:**
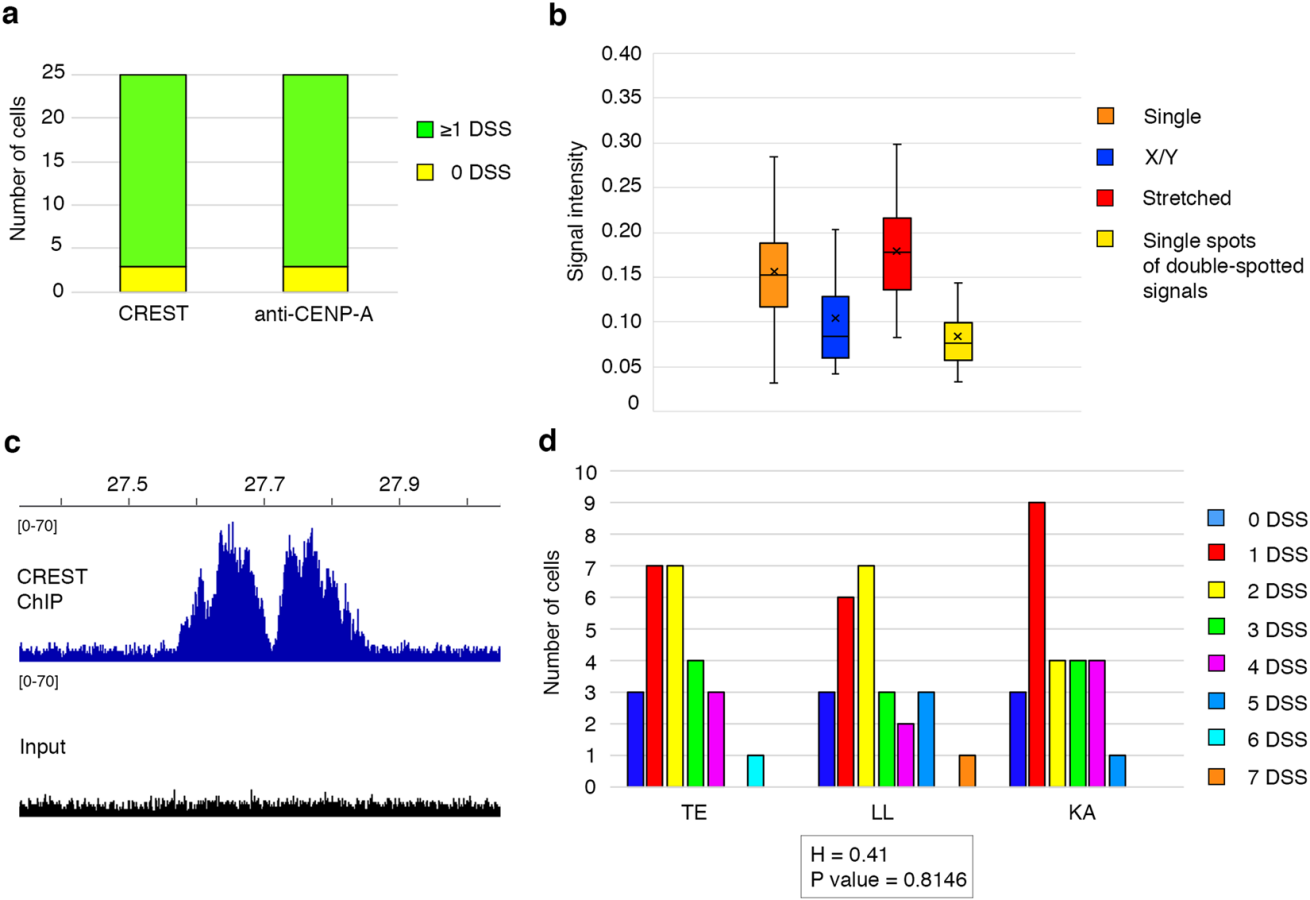
Number, frequency and signal intensity of DSS centromeres. (**a**) Number of cells containing no DSS bivalents (green) or at least one DSS bivalent (yellow) using the CREST serum (left, number of cells = 25) and the anti-CENP-A antibody (right, number of cells = 25) on horse TE. (**b**) Box and whisker plot representing the intensity of signals of single-spotted centromeres (orange), centromeres of the XY body (blue), stretched centromeres (red) and single spots in double-spotted centromeres (yellow). Number of cells = 20. (**c**) ChIP-seq identification of the centromeric domains on ECA 11 in horse TE. Genomic coordinates (Mb) are reported above. Blue peaks represent read coverage from DNA immunoprecipitated with the CREST serum. The dark profile represents read coverage of the non-immunoprecipitated input sample. (**d**) Number of DSS per cell in TE, LL and KA. For each individual, the number of cells with 0, 1, 2, 3, 4, 5, 6 and 7 DSS centromeres are reported. H statistic with P value, derived from the Kruskal-Wallis test, is reported below.

Since the CREST serum may recognize centromeric proteins other than CENP-A, we tested whether DSS are due to the presence of two separated CENP-A domains or to other peri-centromeric proteins that may not colocalize with CENP-A. To this goal we produced an anti-CENP-A serum in sheep immunized with the horse protein. The specificity of this new antiserum was tested by western blotting and immunofluorescence (see Supplementary Fig. S1). We then performed immunofluorescence experiments on horse pachytene spreads from horse TE either with a CREST serum or with the anti-CENP-A serum. Bivalents with DSS were identified in both CREST (Fig. 2a) and CENP-A experiments (Fig. 2b) with the same frequency (Fig. 3a), indicating that they are CENP-A binding domains. In addition, we performed a triple immunofluorescence experiment with the anti-SCP3 antibody, the CREST and the anti CENP-A sera on pachytene spreads. As shown in Supplementary Fig. S2, CREST and CENP-A signals colocalize on all centromeres, including those with DSS signals. These results demonstrate that DSSs correspond to CENP-A binding domains.

The pattern of DSS signals suggests that they correspond to centromeric domains located in different positions on the two homologs. If this hypothesis was correct, the intensity of each one of the double spots should be about half the intensity of single spots. In particular, the intensity of each spot in double-spotted signals should be comparable to signal intensities on chromosomes X and Y, where the two centromeric regions are not paired. We quantified the intensity of all CENP-A signals on 20 pachytene spreads (Fig. 3b). Indeed, the intensity of each dot in double-spotted signals is comparable to the one on X and Y and about half the one on single-spotted centromeres (Fig. 3b). The intensity of “stretched” signals is similar to the one of canonical single-spotted centromeres (Fig. 3b).

Interestingly, no DSSs were observed on ECA 11. To characterize the centromeric domains of ECA 11 in horse TE, a ChIP-seq experiment was carried out with the CREST serum and two adjacent peaks were detected (Fig. 3c). As previously demonstrated^36,37^ the two peaks correspond to the centromeric regions on the two homologs. The proximity of the two peaks in horse TE strongly suggests that they are too close to be resolved as DSSs on meiotic bivalents. The same ChIP-seq characterization of horse centromeres other than the one of ECA 11 is not possible since they contain satellite DNA sequences, which are not assembled in the horse reference genome due to their repetitive nature^35,37^.

DSSs were not observed on ECA 2, whose centromere lacks 37cen repeats but contains other satellite repeat families^27^. To further investigate intra- and inter-individual variability of the DSS centromeres, we performed the same immunofluorescence experiment using the anti-CENP-A antibody on two additional horses, scoring the number of DSS centromeres per cell. From each individual 25 cells were evaluated. Intra-individual variability was high in all the three individuals, with the number of DSS centromeres ranging from 0 to 7 (Fig. 3d) whereas the fraction of cells with at least one DSS did not vary among individuals. The average number of DSSs per cell was 2.0 ± 1.5 in horse TE, 2.4 ± 1.8 in horse LL and 2.0 ± 1.4 in horse KA. Using the Kruskal-Wallis test, we showed that inter-individual variability was not statistically significant (p value 0.8146).

## Discussion

In this work, we describe the chromosomal distribution of recombination events in male horse meiosis. A great inter-cellular variability in the number of crossovers, which ranged from 36 to 63, was observed, in agreement with previous findings in other mammalian species^39–43^.

When considering chromosomal distribution in mammals, it is well known that crossovers are not randomly distributed but their positioning is tightly regulated^5–7^. Particularly relevant is the inhibition of meiotic recombination in the pericentromeric region^15^. This centromere effect has been described in all eukaryotes, including humans and other mammals^12–24^, and we show here that it is clearly observed also in the horse.

We took advantage of the horse model system to test whether the centromere effect on recombination suppression is related to the presence of satellite DNA. To this goal we mapped recombination foci on ECA 11 through the cytogenetic localization of the MLH1 protein along the synaptonemal complex and detected a crossover suppression around the satellite-less ECA 11 centromere, demonstrating that the centromere effect depends on the centromeric function itself rather than on satellite DNA sequences and supporting the hypothesis that recombination suppression at pericentromeres is not related to DNA sequence but rather to the epigenetic environment. It has been proposed that the occurrence of crossovers near centromeres is selectively disadvantageous because it may cause premature sister chromatid separation leading to non-disjunction events at the second meiotic division^24^. It is interesting to mention our recent observation that also mitotic segregation is not affected by the absence of centromeric satellite repeats on horse chromosome 11^44^.

The formation of chromosome bivalents at pachytene stage is accompanied by the pairing of centromeres, as reported for all eukaryotic species studied so far^45,46^. During the analysis of horse meiosis, we observed a peculiar phenomenon: centromeres with double or extended CENP-A signals (DSS) were frequently observed. The morphology of these unusual signals ranged from well separated double spots to long “stretched” signals, which were interpreted as double CENP-A binding domains too close to be resolved separately.

Previous reports on mammalian species, such as the common shrew and the dwarf hamster, detected the presence of double-spotted centromeres^47,48^, although their presence and frequency remained unexplained. In our horse system we identified DSS centromeres at a surprising high frequency on different chromosome bivalents, showing a high inter-cellular variability. In the light of our results, we hypothesized that, at DSS centromeres, the CENP-A binding domains of the two homologous chromosomes are localized on different regions, giving rise to double spots or to stretched signals, depending on the distance between them. This interpretation is strongly supported by signal intensity comparisons in single versus DSS centromeres and the XY body.

The number of DSS centromeres per cell was highly variable (from one to seven) also among cells of the same individual. No statistically significant inter-individual variation could be observed.

The observation of these peculiar centromeres raises the question whether they have any biological meaning. Taking advantage of the satellite-less centromeres of the genus *Equus*, we previously demonstrated that the position of the centromere is not fixed but slides, giving rise to different positional alleles, defined “epialleles”, which are inherited as Mendelian traits^36,37^. Similar polymorphism regarding the position of the CENP-A binding domains was reported also in some human satellite-based centromere, such as the one of HSA 17^49^. Indeed, in HSA 17, the centromere can assemble on different alpha satellite arrays and individuals with heterozygosity in the position of the centromere were reported. A similar characterization is not possible for the horse satellite-based centromeres since their sequences are lacking chromosomal assignment in the horse genome.

**Figure 4:**
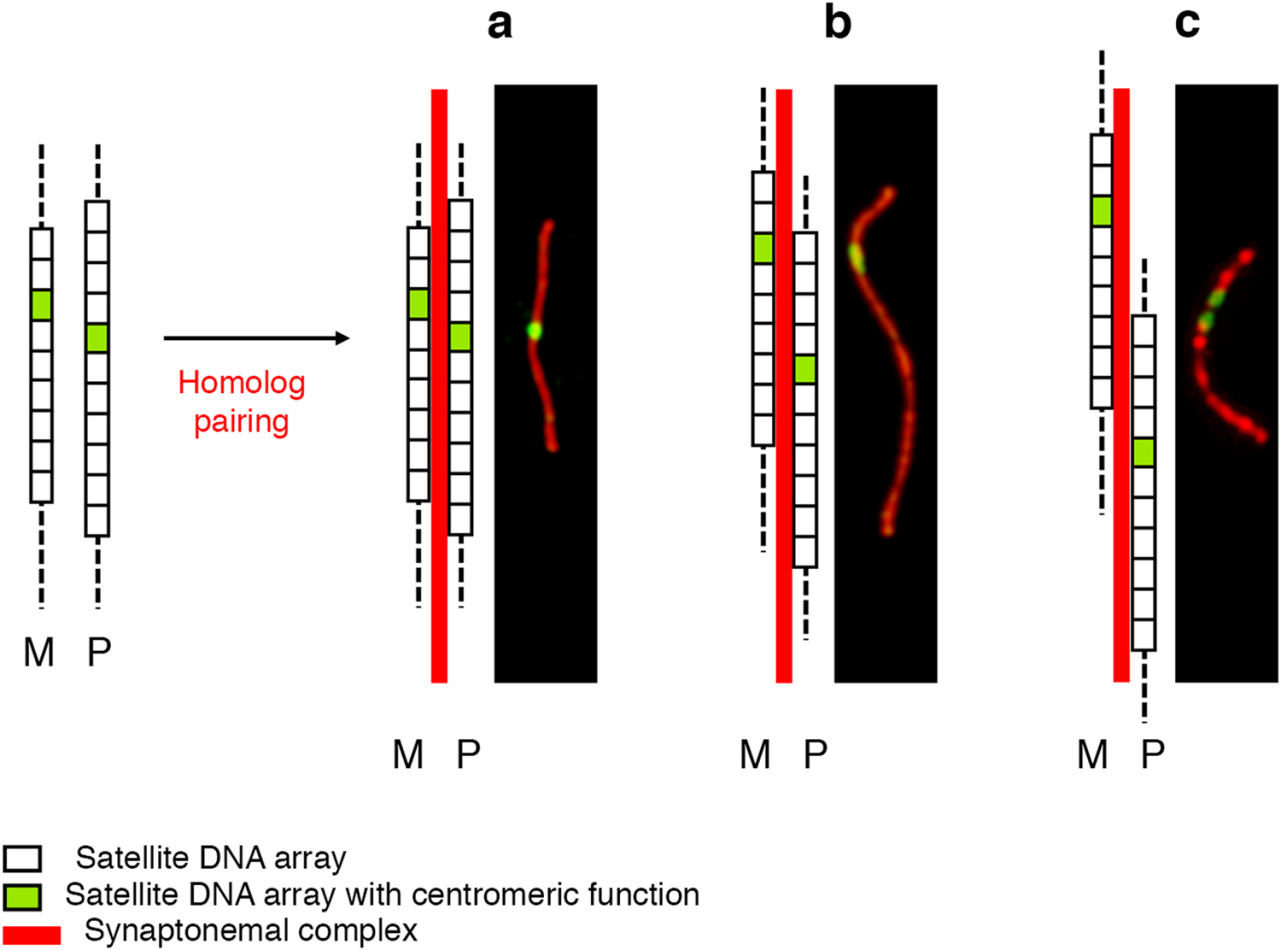
Model for double-spotted centromeres in horse pachytene. On the left, two homologous chromosomes (maternal M and paternal P) with different centromeric position within satellite DNA arrays characterized by different numbers of satellite repeats. On the right, possible scenarios after homolog pairing: centromeres remain too close to be resolved separately (**a**) or become sufficiently distant to be visualized as stretched (**b**) or double-spotted (**c**) signals.

It is known that repeat copy number of satellite DNA arrays can vary, even between homologous chromosomes^50–52^. Therefore, although satellite-less centromeres can only display epigenetic positional variation due to centromere sliding, satellite-based centromeres can also display DNA sequence variation due to variable numbers of tandem repeats. We recently demonstrated that the repeated units of the major horse centromeric satellite DNA, 37cen, are homogeneous at the sequence level^35^. We now propose that the presence of numerous conserved monomers may lead to homology-based pairing between different regions of the extensive array, thus, causing a “staggered” alignment, here called “misalignment”. The presence of polymorphism among homologous chromosomes regarding the position of CENP-A binding domains and the number of tandem repeats suggests a possible interpretation for the occurrence of DSS centromeres. As depicted in the model presented in Fig. 4, we propose that DSSs are the result of two independent components: epiallelism for the position of the CENP-A binding domains and misalignment events between centromeric and pericentromeric satellite DNA arrays of the homologous chromosomes during pairing. This misalignment may increase the physical distance between centromeric domains which may be already in different positions on the two homologs due to CENP-A binding domain sliding. In this scenario, our DSS centromeres would become visible when the distance between the centromeric domains on the two homologous chromosomes is long enough to be resolved by our method (Fig. 4b,c). While epialleles are conserved from cell to cell and inherited as Mendelian traits, the degree of misalignment varies from meiosis to meiosis, resulting in the great variability of the number and distribution of DSSs. Therefore, since we observed that the number of DSSs is variable among pachytene spreads of the same individual and misalignment may affect all satellite-based centromeres, it is likely that all chromosomes could potentially carry a DSS.

Interestingly, DSSs were not identified on ECA 2, whose centromere is the only satellite-based one lacking the 37cen satellite. Although more data would be necessary to test this hypothesis, we propose that the 37cen satellite may be prone to misalignment because its repeated arrays are particularly conserved^35^. The model is also supported by the lack of DSSs on ECA 11 bivalents in horse TE. In this individual, the centromeric domains on the two homologs are very close (Fig. 3c) and no satellite repeat misalignment can obviously occur on this satellite-less chromosome. We cannot exclude that, in individuals in which the two ECA 11 centromeric domains are located on sufficiently distant regions, DSSs may be observed also on satellite-less centromeres.

The proposed model may also explain the observation of a double-spotted centromere in the common shrew and in the dwarf hamster^47,48^. In our system we identified DSS centromeres at a surprising high frequency on different chromosome bivalents, showing high inter-cellular variability. Overall, our findings suggest that the combination of centromere sliding and misalignment of satellite arrays may occur at high frequency in the horse, in agreement with the exceptional centromere plasticity of the *Equus* species.

## Methods

### Testis collection and treatment

Testicular samples from five horses (TE, MP, PV, LL and KA) were obtained by certified veterinarians following castration procedures under general anaesthesia. The castrations were not carried out for our research but were performed as routine management of riding horses. Testicular samples from the five horses were given to us instead of being discarded. All methods were carried out in accordance with relevant guidelines and regulations.

Testes were cut in small pieces (about 1 cm^3^) using sterile scalpel blades and frozen at −80 °C until use.

### Anti-CENP-A serum preparation

For antibody preparation, an *E. coli* codon optimized version of horse CENP-A (ENSECAP00000013849) was synthesized (Eurofins Genomics) and cloned into pDEST17 for expression of an N-terminally 6-his tagged CENP-A protein in E. coli BL21-AI. Inclusion bodies were purified by differential centrifugation, solubilized in 7 M guanidinium-HCl and protein was purified by affinity chromatography on Ni-NTA agarose in 7 M Urea (ThermoFisher). Purified protein was dialyzed against phosphate-buffered saline (PBS) and used as immunogen to raise an antibody in sheep.

### Pachytene spread preparation and immunofluorescence

Pachytene spreads were prepared from frozen testis samples as previously described^53,54^ with minor modifications to adapt the protocol to this horse tissue. Immunofluorescence experiments were performed with the following antibodies: anti-SCP3 antibody (Abcam ab15093), anti-CENP-A sheep serum, CREST serum (kindly provided by Dr. Claudia Alpini, Fondazione I.R.C.C.S. Policlinico San Matteo, Pavia, Italy) and anti-MLH1 antibody (BD Pharmingen, 551091). Fixation with 4% paraformaldehyde (pH 10) in 1x PBS, 0.015% TritonX-100 was used for the preparation of slides for immunofluorescence with the CREST serum. Fixation with 1% formaldehyde, 0.015% TritonX-100 (pH 9.8) was used for the preparation of slides for immunofluorescence with the anti-CENP-A antibody and for sequential immunofluorescence with the anti-CENP-A and CREST sera. The sequential protocol is not optimal for both CREST and anti-CENP-A sera. This is the reason of the sub-optimal immunostaining of centromeres obtained with the combined immunofluorescence.

Slides were permeabilized in 0.05% Tween-20 in PBS. Rhodamine anti-rabbit, Alexa488 anti-sheep, Alexa488 or Alexa647 anti-human and Alexa488 anti-mouse secondary antibodies were used. Pachytene chromosomes were counterstained with DAPI (0.2 μg/ml) and mounted with Fluorescence Mounting Medium (Dako).

### Image acquisition, measurement and statistical analysis

Digital images from fluorescence signals were acquired with a fluorescence microscope (Zeiss Axioplan) equipped with a cooled CCD camera (Photometrics). Pseudo-colouring and merging of images were performed using the IPLab Imaging Software.

Chromosomal length measurements and the analysis of MLH1 foci positions along chromosomal axes were performed using ImageJ 151.s software. The intensity of CENP-A signals was measured, after background subtraction, as Integrated Density, a parameter obtained through the ImageJ 151.s software.

To evaluate inter-individual variability of the number of double and stretched signals we applied the Kruskal-Wallis test using the VassarStats website^55^.

Mean values in the Result section are reported with their standard deviations.

### Fluorescence *In Situ* Hybridization

After immunofluorescence and image acquisition, Fluorescence *In Situ* Hybridization (FISH) was performed as previously described^56^. The 37cen satellite DNA probe was labelled by nick translation with Cy3-dUTP (Enzo Life Sciences) as previously described^57^.

### Cell culture

Horse TE fibroblasts were obtained from testicular tissue after castration. The cells were cultured in high glucose DMEM (EuroClone) medium supplemented with 15% foetal bovine serum, 2 mM L-glutamine, 1% penicillin/streptomycin and 2% non-essential amino acids at 37 °C with 5% CO_2_.

### ChIP-seq

Chromatin from primary fibroblasts of individual TE was cross-linked with 1% formaldehyde, extracted, and sonicated to obtain DNA fragments ranging from 200 to 800 bp. Immunoprecipitation was performed as previously described^35^ by using a human CREST serum^36^. Sequencing and bioinformatic analysis was performed as previously described^37^.

### Accession codes

Raw sequencing data from this study have been submitted to the NCBI BioProject database (http://www.ncbi.nlm.nih.gov/bioproject/) under accession number PRJNA557197.

## Supplementary information

Supplementary information
