# Supplementary material for: CENP-A binding domains and recombination patterns in horse spermatocytes
Source: Sci Rep. 2019 Nov 1;9:15800. doi: 10.1038/s41598-019-52153-1 (PMC6825197; doi:10.1038/s41598-019-52153-1)
Supplement: Supplementary file 1 — Supplementary information [file 41598_2019_52153_MOESM1_ESM.pdf]

Supplementary Information

CENP-A binding domains and recombination patterns in horse spermatocytes

Eleonora Cappelletti[#], Francesca M. Piras[#], Claudia Badiale, Marina Bambi, Marco Santagostino, Covadonga Vara, Teri A. Masterson, Kevin F. Sullivan, Solomon G. Nergadze, Aurora Ruiz-Herrera[*] and Elena Giulotto[*]

[#] These authors contributed equally to this work.

[*] Corresponding authors.

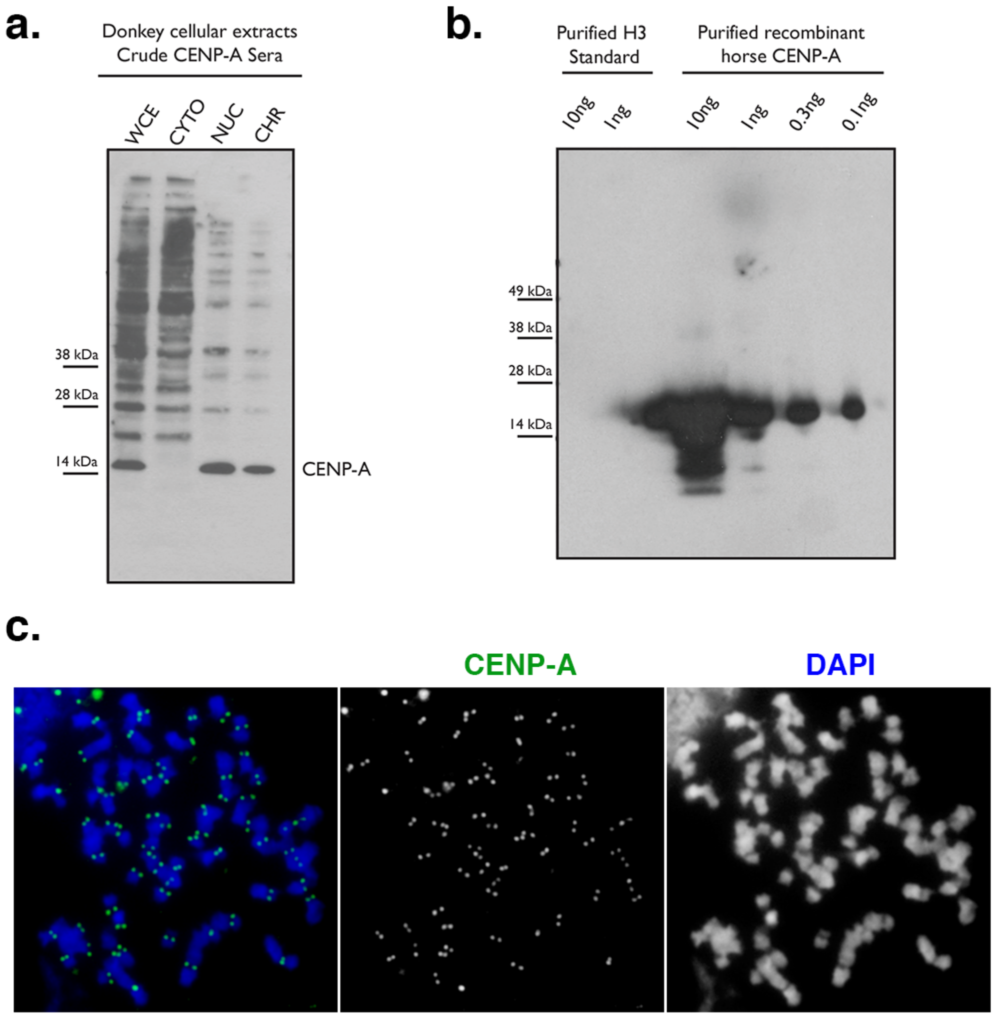

Figure S1. Antiserum raised against bacterially produced horse CENP-A was tested by Western blot (a, b) and by immunofluorescence (c). a) Western blot results against extracts of donkey skin fibroblasts (WCE), cytoplasmic (CYTO), nuclear (NUC) and chromatin bound (CHR) fractions. Antiserum was used at a 1:5000 dilution. b) Specificity of the anti-CENP-A serum for CENP-A versus histone H3. Indicated quantities of bacterially expressed histone H3 (left) or CENP-A (right) were electrophoresed and assayed by Western blot with CENP-A antiserum at 1:5000 dilution. No cross-reactivity with histone H3 was observed. c) Immunofluorescence with the anti-CENP-A serum (green) on DAPI-stained horse metaphase chromosomes from skin fibroblasts. The antiserum labels all centromeres.

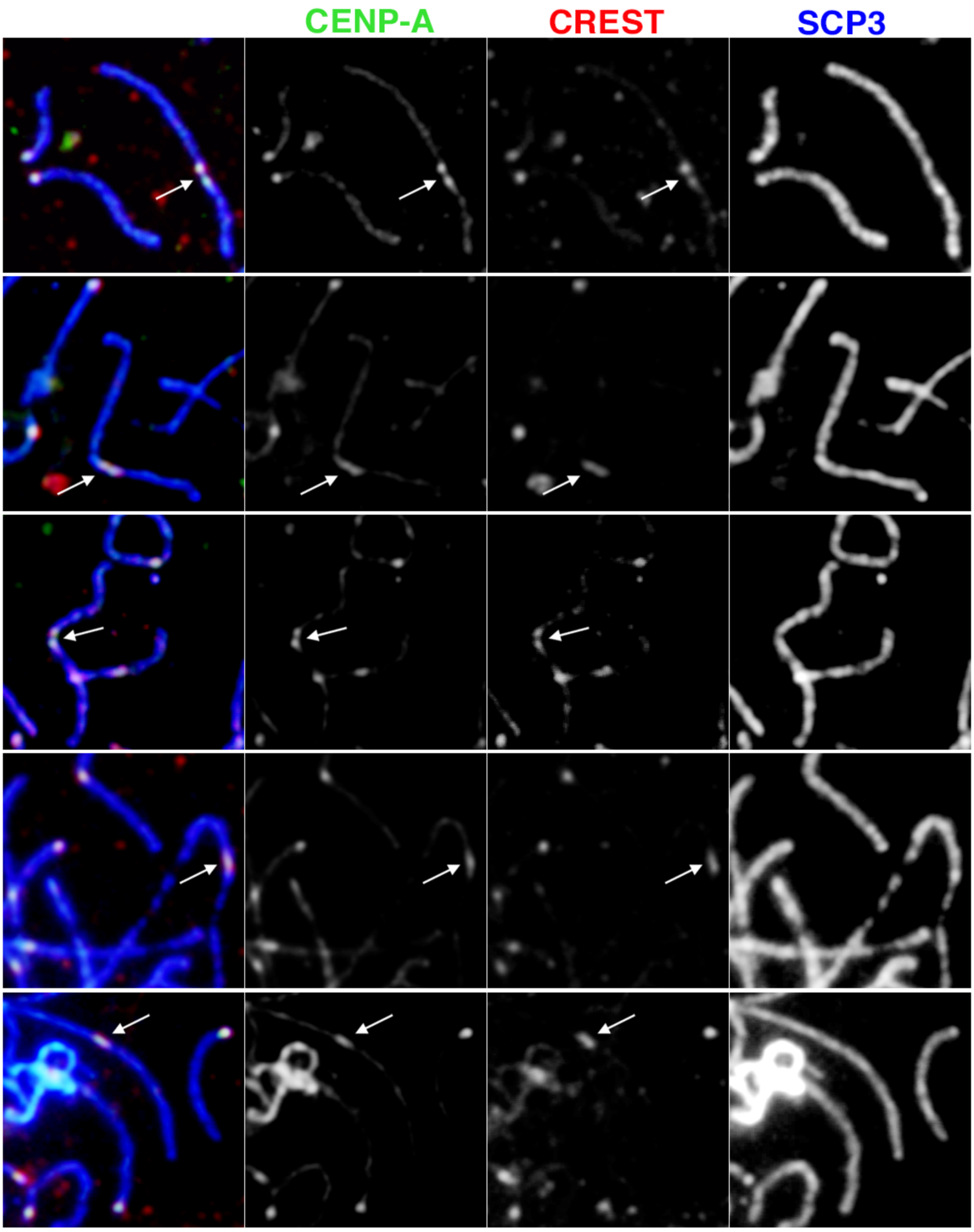

Figure S2. Co-localization of CREST and CENP-A signals in pachytene spreads. Triple immunoflourescence with anti-CENP-A serum (green), CREST serum (red) and anti-SCP3 antibody (blue). The green, the red and the blue channels are shown separately. DSS centromeres are indicated with arrows.
